# Rational Emotive Behavioural Therapy: The Evolution of a Revolution: Interview With Dr. Debbie Joffe Ellis, Work Partner and Wife of Dr. Albert Ellis, the Creator of REBT

**DOI:** 10.5964/ejop.v11i1.911

**Published:** 2015-02-27

**Authors:** Debbie Joffe Ellis, Montse Rovira

**Affiliations:** a Licensed Psychologist, Australia; Mental Health Counselor, New York, NY, USA; b Licensed Clinical Psychologist, Cádiz, Spain

## Abstract

Recognized as one of the most influential thinkers and psychologists, Albert Ellis PhD (1913-2007) revolutionized Psychology when he created the first cognitive psychotherapy, Rational Emotive Behavioral Therapy. After he passed away, Dr. Debbie Joffe Ellis continues spreading his legacy around the world. Psychologist, lecturer, writer, trainer, she dedicates her life to disseminate REBT and extend it through different statements, from the social to the educational, from the academic to the clinical. In this interview, she goes through her own history and her husband’s one, bringing us closer to understanding Albert Ellis as the leading figure in his field, and the oneness they experienced through their professional and personal relationship.

## New York - January 2014

If next winter is not as cold as the last one, New York citizens will remember January 2014 as the iciest in decades. While the taxi was driving me from the airport to the City through a snowstorm my horizon was limited for the back part of the car that was ahead. We were going slowly through a viscous fog that made blurred the skyline of Manhattan. I was going to interview Dr. Debbie Joffe Ellis, partner and widow of Dr. Albert Ellis who revolutionized the Psychology.

## Interview

Dr. Debbie Joffe Ellis is an enthusiastic woman. She is passionate about her work and about her beloved husband. I had seen them in the pictures looking at each other with pleasure, always smiling, holding their hands. I wanted to know who that woman was and is.

**Montse Rovira**: Who is the woman holding Albert Ellis’s hand?

[Contrib contnr1]**Debbie Joffe Ellis**: The woman who holds Albert Ellis’s hand is a human being, who admires the scientist, respects the person and loves the man. Al and I shared the same values and the same interests. There was a huge age difference and yet it was irrelevant. We had a similar sense of humor. We loved helping other people and teaching them how to help themselves. We loved teaching and writing. We loved spreading REBT so that people could learn how to help themselves. We both were passionate about one another. We believed in what we did. We shared study and work. We shared life.

**Montse Rovira**: “To Debbie Joffe Ellis, the greatest love of my whole life, my whole life”. Your husband and you shared work and life. What can you tell me about the doctor and the man?

[Dr. Joffe Ellis’s eyes shine when I remind her how Dr. Ellis dedicated his autobiography to her (Ellis, 2010, *All Out! An Autobiography*). She looks at me in a caring way, breathes softly and smiles.]

**Debbie Joffe Ellis**: My husband was well known for many things. For changing the direction of Psychology, for pioneering Cognitive Psychology, for his 85 books, for 1.500 plus articles, for running groups all over the world teaching, etc. He is known also for being a pioneer in the area of relationships and sex. He was one of the first people in the 1940’s and onwards to fight for women’s and gay’s rights, and it wasn’t so popular then! So he became an expert in relationships and love and sex and he wrote about that. One of his most famous books on this topic is “Sex without guilt”. Nowadays he’s recognized as a major contributor to the Sexual Revolution in America, which spread to other countries. Al was brilliant, controversial, innovative, but besides all of that, he was an extraordinary man.

Al loved what he did. His work was his mission. He typically worked 18 hours or more each day. He loved his work. He loved love. He loved women. He was married the first time (for 18 hours!), but they stayed friends for the rest of her life. Then he married again (that lasted 18 months!) and after they divorced still continued their friendship. He had relationships with many women. The relationship that Al and I shared was something very rare, unusual, a very, very remarkable circumstance. My husband told me many times that he had not felt that depth of love in any of his previous relationships that he felt with me and from me. It was unconditional love.

[Dr. Joffe Ellis sips from her coffee and her sight loses in time.]


**Figure:**
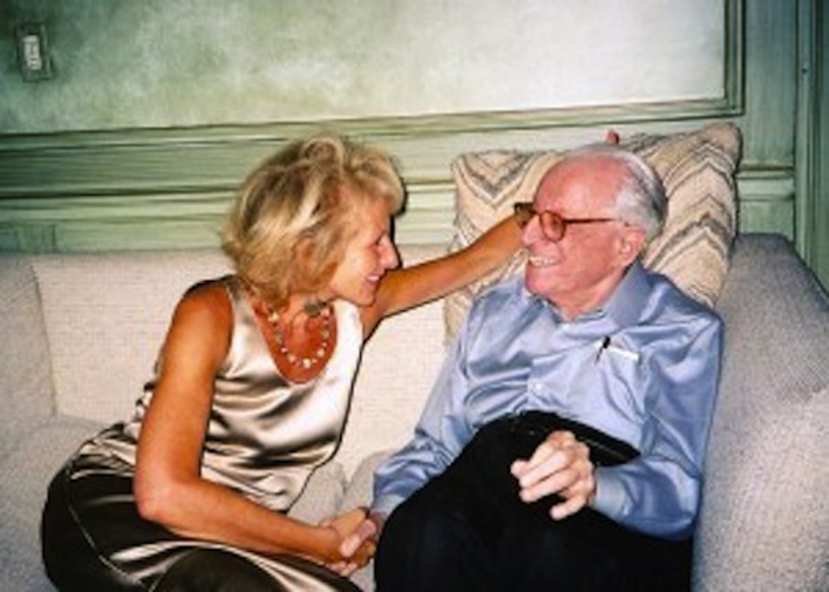
“I first met Al in my aunt’s library”


**Debbie Joffe Ellis**: As you may know, I was born in Australia and raised there. My aunt was a psychologist and had a huge library. I loved reading and was strongly interested in Psychology. So, I would frequently visit her home and that library was an enigmatic and surprising place for little Debbie. I looked at all those books and asked my aunty about them. It was such a temple of wisdom and each book was a treasure. I distinctly remember that one afternoon a book caught my eye. Its name was “Reason and Emotion in Psychotherapy”, by Albert Ellis. I took it and read some paragraphs and they deeply impressed me. So it can be said I first met him in my aunt’s library!

Then some years later, I studied Psychology, and during that time he visited Australia and gave workshops at the University I studied at, University of Melbourne. I saw him there. We spoke briefly, and I thought, “Wow! Albert Ellis!” Never did I think that one day in the future I would get married to him. Then we met again, maybe 15 years after that, in San Francisco, at the APA Annual Convention, and we started our friendship. Profoundly close. I went back to Australia and we wrote to each other frequently. We spoke by phone once or twice each month. Then I started visiting New York once a year.

[Dr. Joffe Ellis’ sight gets darker before continuing with a quiet voice:]

**Debbie Joffe Ellis**: I was in New York in 2001 when World Trade Center was attacked, and I decided to make longer my stay. I volunteered in a place, which was opened 24 hours a day, and recovery workers and policemen, and firemen could come there anytime to get some food or drink. I would sometimes serve the food or clear the tables, and would mainly be there to listen to and to talk to the people. Many of them were in a strange emotional zone of stoicism for getting the work done, looking for bodies, dealing with seeing shocking things like body parts or burned toys of children, and people's shoes and torn clothing and other things that were so upsetting. As the weeks went on, they were more exhausted at times. For some of them shock had changed to anger. I did not sit and give formal "therapy", but would be there for them, be with them, listen and care, and encourage any who wanted to talk to share, and when we found it appropriate we would talk about ways they could feel less angry or depressed or overwhelmed.

During that time, I was also helping my husband who was working with little rest. Several clients wanted to change their session’s in-person into phone ones because they were afraid of taking public transportation. We still gave our regular Friday Night Workshops, and he also gave a talk on terrorism, discussing with the audience on what REBT could do to help people and possibly reduce their rage and panic and fear about terrorism. They were very intense days and we were working hard together. And we loved doing so. We were inseparable after our relationship began. Our bond became stronger and finally we married. We had an enormous amount in common. We cherished life. I still do. I am grateful for my life and the good things in it. What brings me a great joy is to contribute to others, and I find very gratifying for me when I give presentations, and workshops and demonstrations. Like Al, I find happiness helping people to discover happiness through REBT and its tools.

**Montse Rovira**: And if I would not know about REBT and its tools… What would you explain me about it?

[Dr. Joffe Ellis smiles and showing her training abilities explains:]

**Debbie Joffe Ellis**: REBT stands for Rational Emotive Behavior Therapy and it is the pioneering cognitive approach in Psychology and Counseling and Therapy. My husband was really the first one to challenge Freud in the nineteen forties and the nineteen fifties. At that time – at least in America – Freud was the “God” of the Psychology universe or Psychiatry. My husband studied at Columbia University and at that time, he had no choice but to study Freud and Psychoanalysis and to practice it with people. And as he did that, he noticed that some people were feeling better but they were not getting better, they were not getting to the root of the issue in order that they could stop creating their emotional suffering and start practicing healthy thinking and behavior.

So more and more he became active-directive with his clients, and he would give them homework and he would give them the idea that they were responsible for creating their own emotions. They discussed what was creating emotional disturbance and was unhelpful to get their goals. So bit by bit, he was developing his approach in what in the beginning was called Rational Therapy, after it became Rational Emotive Therapy and in 1993 he put in the “B” for behavior: REBT.

**Montse Rovira**: He founded REBT, which was a revolution in the field of Psychology and Psychotherapy breaking with all the classical approaches, isn’t it?

**Debbie Joffe Ellis**: Yes. Al established the premises of a new therapeutic model that broke with the traditional work between client and therapist, with the classical approach to mental and emotional disturbances and with the methodology to solve them. An essential aspect of the cognitive revolution pioneered by my husband is: “It is not what happens or outer circumstances that create our emotions, but the way we perceive those events and circumstances”.

**Montse Rovira**: What would you in your role as psychologist do with me if a suicide pilot crashes his plane into a building and kills my loved ones?

**Debbie Joffe Ellis**: The approach that I would use with you would be similar to the one I would use with anyone losing loved ones in both such tragic – and also non tragic – circumstances. Grief is grief, whether it has added to it shock from unexpected and brutal death of loved ones, or their death after a long illness when it is not unexpected. I would provide listening, empathy, caring. At an appropriate time, we would discuss the fact that death is an inevitable part of life, and though we are deprived without our loved ones, we can choose to focus on our love of them, and to also focus on what still is good in our lives.

[Suddenly I realize that these words are pronounced by a woman who lost her beloved husband, and more recently her mother, and yet she makes the effort to focus on the good things she still has in her life.]

**Debbie Joffe Ellis:** Your negative emotions can be unhealthy like depression, or healthy like sadness. We would distinguish between appropriate sadness and grief, which would be healthy, and depression, which would not be healthy. REBT reminds us that we have choice. We can choose to feel the depth of our loss, which is not depression, and we can choose to accept what we cannot change, and remind ourselves of the importance of self-care: choosing to live full lives, cherish life, as all of us will die someday.

When we are willing to teach ourselves to think in healthy ways, we create healthy and appropriate emotions. When we are thinking in unhealthy ways, irrational ways, then we create debilitating unhealthy emotions, for example: anxiety, panic, depression, jealousy, shame, rage, guilt, and so on. We create these emotions through our irrational way of thinking. So REBT helps people learn that they have control of their emotional destinies. Al was a visionary, an idealist, and he was also a realistic. He reminded people that life passes quickly, and to enjoy what we can in this life, that life will include loss and suffering. But when we think in healthy ways and create healthy emotions, we minimize the suffering. We cope with it better, we are happier more of the time, and in doing so we can, – even if we are not psychologists –, help other people and create a healthier community, a healthier society, and a healthier world. That was his ideal.

**Figure:**
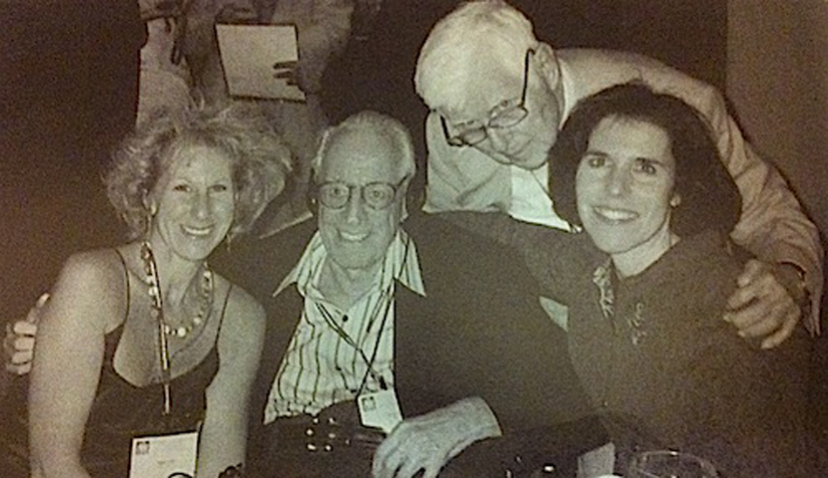
“You have to be a detective and find the thoughts that are hurting you”

**Montse Rovira**: Are appropriate thoughts the key to achieve our mental and emotional balance?

**Debbie Joffe Ellis:** Healthy beliefs create healthy emotions, healthy emotions and healthy beliefs contribute to healthy behavior. They are all interlinked. One of the things I love about REBT is that it is so holistic, it is not just about the mind. It recognizes mind, behavior, emotions and body are all interconnected. Another thing I love about REBT, which makes it unique, and sets it apart from the many other cognitive approaches that followed it, is its philosophical emphasis. And the emphasis on having compassion for ourselves and for others. And its vigorous push to practice unconditional self-acceptance, unconditional other acceptance and unconditional life acceptance. The other cognitive approaches would not disagree with that, but they don’t emphasize it in the same way.

[Dr. Joffe Ellis is very expressive. Her corporal language is as expressive as her words. She sounds like she is really firm and convinced. She continues:]

**Debbie Joffe Ellis**: And the other thing I love so much about REBT is its precision, it does no just say change your thinking, it says look at your thinking, be a detective, be a scientist, you’ll find the thoughts that are hurting you. And don’t just change them, but first identify them, then dispute them, get them from their roots, get rid of them and then start thinking in healthy rational ways. It is more precise and specific, we are urged to identify the “should’s and the must’s”, and the precision of the disputing phase is emphasized more than in many other cognitive approaches.

My husband gave credit to the people who influenced him. Some of them were the ancient stoic philosophers, Greek and roman philosophers of classical times, eastern philosophers, Lao-Tse, Confucius. Moving forward to my husband’s lifetime, contemporary philosophers of his time which include Bertrand Russell, John B. Watson, John Dewey and others. Also Alfred Adler, and his approach of Individual Psychology. My husband had great respect for Adler and gave credit to him for being an influence. Adlerian psychology includes some cognitive approach.

**Montse Rovira**: What about Aaron T. Beck, well known as the founder of Cognitive Psychology?

**Debbie Joffe Ellis**: Of course! Tim Beck and my husband had a respectful relationship. Beck is younger than Al, but if you look in my husband’s autobiography you will see some of their correspondence. Beck sent some of his early papers and ideas to my husband, and my husband wrote back in detail every time. Beck’s cognitive approach came ten to twelve years after REBT. Historically REBT was the big pioneering approach and CT and CBT came later. The main differences between them are the philosophical emphasis of REBT, the emphasis on unconditional acceptance seen in REBT; and the precision of disputing irrational ideas that REBT has. In the nature of REBT and my husband’s style, many people say that there is more vigorous energy than may be seen in the other cognitive approaches. I’m not saying which is better or worse, they are similar but different. Both recognize the great power of cognition.

[During the last years the technological advances have allowed us to study the brain with techniques that were unthinkable just two decades ago. To understand how the brain works it is necessary to study it in vivo, to check which areas get activated in their answer to certain stimuli. See what happens on the neurological level when we act, feel or think in a certain way. Disciplines such as Psychoneuroinmunnoendocrinology, Biopsychology or Neuropsychology, have developed exceptionally thanks to technology, and there are more and more labs focused on brain research.

I try to imagine which difficulties could find Dr. Albert Ellis seventy years ago when he decided to quit with classical psychotherapy and he started talking to his clients about thoughts, reason, consciousness… in a time when those terms would be considered such as “paranormal” concepts. I tell my reflections to Dr. Joffe Ellis. She nods and smiles:]

**Debbie Joffe Ellis**: Many people don’t know that they have a choice in how they feel. Some demand help from a therapist without realizing that they can themselves create changes in their feelings and thinking after they learn how to do so. A good therapist explains the reasons why they feel badly and teach them the tools to practice for the creation of healthy thinking and feeling and behaving. My husband realized that when we are aware of our irrational, illogical and unhelpful thoughts we have taken the first step on the path to achieve the change, but we have to be persistent in our efforts. Being aware of our irrational beliefs is the first step. Then comes the disputing, followed by the creation of effective new ideas and beliefs.

**Montse Rovira**: Several researches on neuroscience assure that a high percentage of mental functions are unconscious. How can we dispute and change our automatic thoughts if we are not aware of them?

**Debbie Joffe Ellis**: Here is the thing! You see, REBT is mainly focused on the present. On what is the disturbing emotion now, in your life at this time that you want to change. That’s what we start with, you know, with a goal of greater happiness in life and less unnecessary suffering, which we create. No matter what happened in the past, REBT focus on the present. And then we do the detective job, we look for the “B”, the beliefs. And we dispute them and come up with new healthy beliefs and philosophies. So it works on your conscious beliefs. We know there are unconscious processes, but the unconscious has not been identified in a concrete way, or as a function centered in a particular area of the brain. Maybe it is a processing working way of the brain itself. People who believe in the unconscious probably would say that conscious beliefs are influenced by the unconscious, and essentially we work with these conscious beliefs.

**Montse Rovira**: I am not sure of being aware of all my thoughts.

**Debbie Joffe Ellis**: I will give you an example. Some therapists do dream work, they analyze them. Our dreams are the outcome of an unconscious process and we usually don’t manipulate them. Ok, there is no problem! Because when you are doing dream work you are consciously describing the dream you had. So you may say to me “I dreamed I was falling down from the Empire State Building”. And so, what was the emotion that you felt? And you might say: “Terror, anxiety, panic!” And I might ask: Well, what do you think you might have been telling yourself to create your terror, anxiety and panic? “Oh! I shouldn’t be falling like this. Oh! I’m going to die, that’s terrible!” Can you see? You can even use dreams to stimulate the discussion and identify some of the irrational beliefs. So let’s ask: have you ever had some of these irrational beliefs in your conscious life? You see? REBT is not afraid of anything. It can be used in any situation, to look at any emotion, any thought, no matter wherever it comes from. A session starts from the present and may look to create healthy goals for the future. If you change your cognition, emotions and behaviors will also change.


**Figure:**
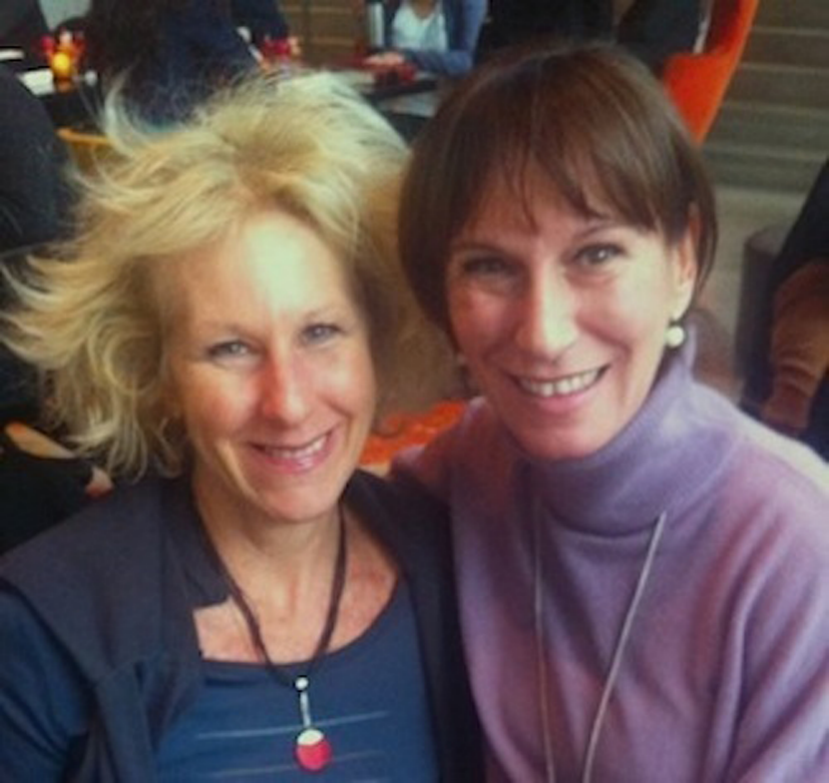
“When we change our mind, we also change our brain”


**Montse Rovira**: When I change my way of thinking, am I also changing my brain?

**Debbie Joffe Ellis**: Correct! Yes, indeed! You are talking about neuroplasticity. You know, when my husband created REBT in the nineteen forties there was no technology to study the brain like there is now. This word, “neuroplasticity” it didn’t exist, although some scientists like Ramón y Cajal did talk about functional plastic changes in neurons. My husband was ahead of his time in so many ways!

[Dr. Joffe Ellis looks at me with complicity:]

**Debbie Joffe Ellis**: I’m going to tell you something that some people may not know. Al was member of Mensa, an international organization which condition of being a member is having exceptionally high IQ. Al had an exceptionally gifted and brilliant mind, his IQ was in the highest percentile of IQ along with the likes of Einstein. But let’s go back to neuroplasticity. Al gave people homework and said “do it for thirty days”. He had the intuition that this was an appropriate period to install new habits, both behavioral and mental ones. Now research supports that 30 days is a good period of time to affect the synaptic behavior in the brain. Today that’s obvious, but for him it was only a supposition, an intuition, a vision. New cognitive habits really create new mental routes, brain’s morphology changes. If we are persistent these new routes become stronger, and the old ones weaken or are extinguished. A very good example is that person who says “I’m a new man, completely different than I used to be”. In fact, it is the same person with new brain circuits, more rational. This rational way of thinking provides healthy emotions and effective behaviors to achieve goals. Isn’t it prodigious?

I could say the same about Mindfulness. Research now supports the effects of Mindfulness and focus. Al talked about Mindfulness sixty years ago, when that term was rarely used in Western society. Al recommended mindfulness as homework because he knew it would be helpful to get the routine of focusing on the present and being aware of our thoughts.

Al had a huge sense of humor. This is another of his unique qualities which he integrated into his methodology. He loved to write lyrics of songs and in his workshops, he and attendees together would sing his Rational Emotive Songs. Singing and music stimulate some parts of the brain. So yes, he didn’t call it neuroplasticity, the research wasn’t there, but he was already encouraging principles and actions which affect the psychology and physiology of the brain.


**Figure:**
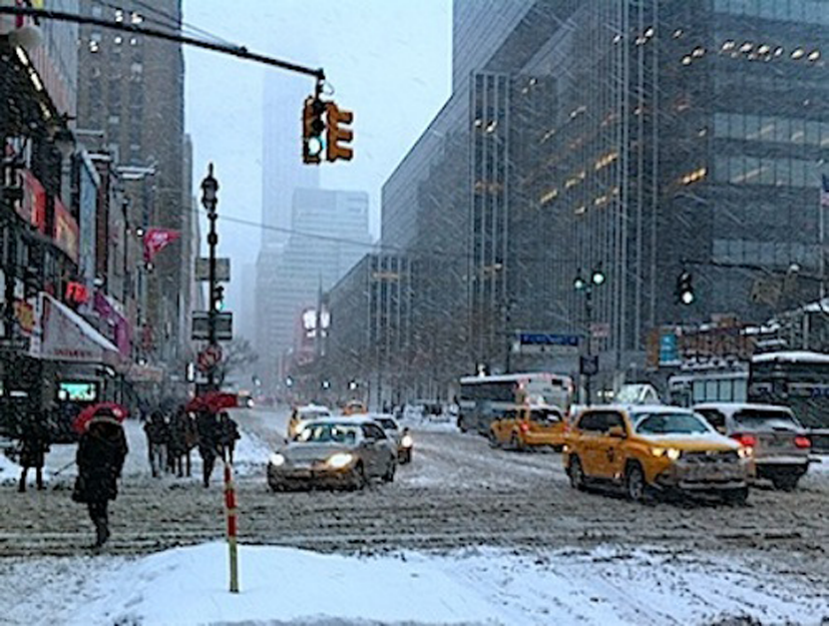
“It is irrational to think that everybody will always act in a rational way”


[Time is passing while we share coffee and conversation. The frontal wall of the bar is a large panoramic crystal that brings us a wonderful view of the Lincoln Center surroundings. We look outside and realize that it is getting dark.]

**Montse Rovira**: It is surprising to me that the City is hardly crowded. Almost there are no pedestrians and no traffic on the street.

**Debbie Joffe Ellis**: It is because of the weather tonight. The temperature itself is no so low but the sensation is about -13ºF due to intense winds, and that is extremely cold for New Yorkers, even local people here may not be used to it. Meteorologists advised people only to go outside if it was urgent, especially after the sunset. But this is only a very little adversity! Remember: if you are alive, no matter how bad things are, they can always be worse! You and me are now here, in a lovely place, sheltered from cold, sharing a coffee and a nice conversation. We can choose to enjoy it or we could also start complaining about how cold will be outside when we go back home. It is so much better if we focus on this delicious present moment. Extrapolate this insignificant example to any circumstance in your life.

You have the choice to focus on what you want. Do it always on the good things that your life offers to you day by day, feel grateful about them. This is the way of thinking that my husband preached and he continued to remind everybody. He was an example of rational thinking and living. With that way of thinking, he could overcome his fear of public speaking, or his fear of rejection from women that he had suffered from when very young. Al practiced REBT on himself and encouraged others to take healthy risks at times, even if afraid to do so. Al was very, very assertive with his clients: “Do it! And if you die, you die! We will give you a very nice funeral!”

**Montse Rovira**: He was convinced about human capacity to transform, about our own potential.

**Debbie Joffe Ellis**: He was a visionary ahead of his times. However, we have the potential to transform ourselves, to be extraordinary in terms of not succumbing to the easy tendency to make ourselves anxious or depressed, for example. We can think of him as a model for doing so. We are fallible humans, and sometimes we may continue to think and act in an irrational ways, as can those who we have relationships with. It is irrational to think that everybody can always act in a rational way. People can significantly change their lives when they don’t give up making effort to do so: that is what Al wanted to transmit. Don’t give up. Love, laugh, be grateful, cherish your life, accept what cannot be changed, put your sight in the present and have healthy goals for the future.

[In my mind emerges the idea that we perpetuate our emotional disturbance when obstinately live in a wrong time, maybe in a distant perfect past or a conditional future. I stop the recorder and an intruder thought invites me to wonder if I’m going to see Dr. Debbie Joffe Ellis again, but immediately I say to myself that that’s an unhelpful question, and absurd way to concern about the future. I come back to the present and tell her the only words that come up my mind: “Thank you Debbie”.]

## Epilog

Six decades have passed by since REBT was presented to the American Psychological Association. Dr. Joffe Ellis has explained through her professional and personal relationship with Dr. Albert Ellis, how REBT was originated, which were the main theoretical sources of it, its methodological bases and its protocols. How it has been developed from an initial version focused on cognitive processes to the holistic dimension that it already has, taking into account cognition, emotions and behavior as inherent aspects of human being, which interconnection has to be considered as the main goal of psychotherapy. The pioneer cognitive overturn that Dr. Albert Ellis gave to Psychology has developed into a “School of Thinking” and its influence goes beyond the strictly clinical field.

For further information see: www.debbiejoffeellis.com and Ellis and Ellis (2011). For a Clinical Demonstration of REBT: see the DVD, released in 2014, by Debbie Joffe Ellis: Rational Emotive Behavior Therapy, produced by the American Psychological Association, Washington DC (Ellis, 2014).
